# “The Critical Services Are Out of Reach”: Diabetes Management and the Experiences of South Asian Immigrants in Ontario

**DOI:** 10.1177/21501319241240635

**Published:** 2024-03-24

**Authors:** Minal Waqar, Vincent Z. Kuuire

**Affiliations:** 1 Department of Geography, Geomatics & Environment, University of Toronto – Mississauga, Mississauga, ON, Canada; 2 Behavioural Health Sciences Division, Dalla Lana School of Public Health, University of Toronto – St. George, Toronto, ON, Canada

**Keywords:** South Asian, diabetes mellitus, health services utilization, Canadian healthcare system, migrant health

## Abstract

Type 2 diabetes is a serious chronic condition affecting millions of people worldwide. South Asians (individuals originating from Pakistan, India, Bangladesh, Sri Lanka, and Nepal) represent a high-risk ethnicity for developing type 2 diabetes (T2D) and experience a high prevalence of the disease, even in migrant populations. The objective of this study was to investigate perceptions and experiences of South Asians living with T2D in Ontario, and their utilization of diabetes related services within the provincial healthcare system. Data were obtained from 20 in-depth interviews with South Asian participants diagnosed with T2D and living in the Greater Toronto Area. Our findings indicate a dissatisfaction with Ontario’s coverage for diabetes services; varying uptake of recommended health tests, exams, and monitoring equipment; low utilization of additional resources (diabetes centers); and a need for primary care physicians to better facilitate awareness and utilization of available coverages and resources in the community. This study provides support for the fact that even in Canada’s universal healthcare system, disparities exist, particularly for ethnic minorities, and that a universal prescription drug coverage component is a crucial step forward to ensure equitable access to health services utilization for all.

## Introduction

Type 2 diabetes accounts for the vast majority of diabetes cases (90%) around the world.^1,2^ The global prevalence of type 2 diabetes (T2D hereafter) has been increasing at an epidemic rate, having nearly quadrupled between 1980 (108 million) and 2014 (422 million).^1^ South Asians, that is, individuals originating from Pakistan, India, Bangladesh, Nepal, and Sri Lanka, represent a high-risk ethnicity for developing diabetes, and they also face disproportionate diabetes-related complications and mortality rates.^3^ Past literature has clearly demonstrated that migrant South Asians living in Western countries experience 2 to 4 times the rate of being diagnosed with diabetes compared to the general Caucasian population.^4-6^ Moreover, South Asians are prone to developing T2D at a lower BMI and younger ages compared to other ethnicities (including Caucasians). Existing literature has also established that the onset of diabetes can occur up to a decade earlier among South Asians than white European counterparts.^7-9^ Consequently, South Asians also have a higher risk of developing diabetes-related complications (nephropathy, retinopathy, and peripheral neuropathy) compared to the host population.^7^

Within Canada, the South Asian population is a migrant population, and as such, it forms an integral part of Canada, accounting for 7.1% (2.6 million) of the country’s total population.^10^ South Asians also represent the largest proportion of Canada’s visible minority population, followed by the Chinese and Black population.^10^ Thus, the wellbeing of such a considerable proportion of individuals carries significant implications, extending beyond the healthcare system to the broader economy, as well as other high-risk T2D populations within Canada.

While most immigrants commonly experience the “healthy immigrant effect” upon arrival into host countries, this effect diminishes over time, leaving migrant populations at an increased risk for declining health outcomes.^11^ This decline occurs primarily due to acculturation in the host society, that is, uptake of unhealthy lifestyles such as smoking, drinking, and physical inactivity; exposure to environmental factors; and lack of experience with a new healthcare system.^11,12^ It is also worth noting that successful acculturation is partially dependent on integration into the workforce, however, a majority of the immigrants face credential recognition barriers imposed by Canadian employers which impede their participation in the labor market.^13^ Additionally, new immigrants often face difficulties in the use of health services and are less likely to benefit from prevention or treatment strategies, partly due to language and cultural barriers, and partly due to insufficient information or misinformation, leading to non-compliance to treatment, lack of awareness on ethnic differences in health, and eventually a decline in overall health status.^12,14-16^ This decline in the healthy immigrant effect, combined with an existing predisposition subsequently places some ethnic groups at an elevated risk for developing T2D, including South Asians.^16^

Despite the ample literature on the challenges that migrant South Asians encounter in accessing healthcare services in Western nations like the UK, USA, and Canada, there remains a research gap in examining the tangible utilization of health services related to diabetes (eg, eye exams, blood tests, foot exams, medications, equipment, and community resources) among South Asians, and even more so in the Canadian context. As a result, the objective of this study was to investigate experiences of South Asians living with T2D and their utilization of diabetes-related health services within the Canadian healthcare system (specifically Ontario). Drawing on ideas from Andersen’s Behavioral Model of Health Services Use^17^ and the relational perspective of place on health,^18,19^ we conceptualize utilization of T2D services as complex dynamic events, which are influenced by individual and contextual factors concurrently operating at various scales with consequences for health outcomes. The findings hold notable importance in comprehending the utilization of health services related to diabetes, and strongly make a case for policy changes geared toward enhancing access to health services, benefitting not just South Asian patients, but also encompassing all individuals affected by T2D.

## Health Policies Governing Access to Healthcare in Canada

Canada has a publicly funded healthcare system that is mandated at the federal level and executed independently at the provincial and territorial level. Provincial and territorial governments have primary jurisdiction in healthcare delivery and administration, but they still operate under national principles that must be reflected in their healthcare insurance plans.^20^ These national principles were defined by the federal government in the *Canada Health Act* (CHA), which was introduced in 1984. The aim of the CHA is to warrant that medically necessary services are available to all Canadian residents, regardless of their ability to afford or pay for those services. The CHA sets out 5 main principles for the provinces and territories to follow in order to receive federal funding for healthcare: public administration, comprehensiveness, universality, portability, and accessibility.^20^ As of April 1, 2020, medically necessary diagnostic services for example, MRIs and CT scans were also added as fully insured services to the CHA.^20^

Although the CHA seemingly removed direct costs of receiving healthcare and promoted standardization in the funding of insured services across provinces, recent research has reported a trend of increasingly more Canadians encountering accessibility problems.^21,22^ These difficulties range from financial barriers to organization of services to accessing appropriate primary healthcare services in Canada, with most of these obstacles being encountered by specific subsets of the population, such as immigrants.^21,22^

Another major shortcoming of the CHA is that it excludes universal coverage for prescription drugs (referred to as a national pharmacare plan) used outside of hospitals.^23^ While the provinces and territories allocate limited subsidies for prescription drugs, the majority of the costs are left to the patients to be paid out-of-pocket or through private insurance.^23^ This is a glaring hole, especially in the “accessibility” principle of the CHA, because prescription drugs can manage, prevent, and even cure illnesses, so they form an integral part of medically necessary care.^23^ This issue affects all Canadians, but the most heavily impacted are those with lower income backgrounds and those without private insurance.

At the provincial level, that is, in Ontario, 2 health policies are relevant in governing access to healthcare. The first is related to the prescription drug coverage or lack thereof—Ontario Health Insurance Plan (OHIP), which does not cover any prescription medicines for individuals aged over 24 and under 65 years, and they have to commonly rely on either of 2 drug coverage plans: Trillium Drug Program (partially covers high prescription drug costs according to family income) and the Ontario Drug Benefit Program (covers prescription medicines for individuals aged over 65 years, or long term care patients, or those who get social assistance).^24^ The second policy pertains to new permanent residents (NPRs) or landed immigrants in Ontario, including economic skilled immigrants, family-sponsored immigrants, and refugees, who were formerly required to undergo a 3-month wait period in order to become eligible for provincial healthcare coverage or OHIP. This waiting-period requirement was recently removed by the province in response to the COVID-19 outbreak as of March 2019, thus granting all new eligible and returning residents immediate health insurance coverage.^20^

Aside from these federal and provincial level health policies, there are a number of important factors that influence access to healthcare for immigrants and/or ethnic minorities in Canada: lack of cultural sensitivity in service provision, especially in terms of understanding and communication between patient and provider; adequacy of information; language and literacy difficulties; and financial barriers involving health insurance and out-of-pocket costs.^20-27^ However, it is important to note that the literature available on access to diabetes healthcare services far outweighs the research available on the actual utilization of such services.

## Framework for Understanding Health Services Use

**Figure 1.**
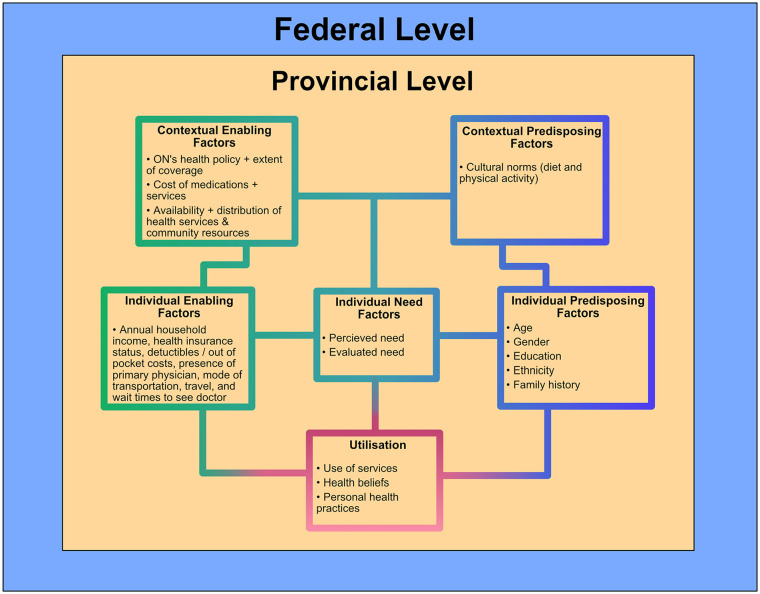
Conceptual framework showing a combination of factors from Andersen’s BM^17^ and the relational perspective of place in health. The factors shown have complex and constant interactions which are in turn embedded within the provincial and federal scales.

Among the theoretical frameworks for understanding health care services use, the Andersen’s behavioral model (BM) of health utilization is arguably the most popular. The model posits that an individual’s utilization of health services is determined by the interactions between predisposing, enabling, and need factors, which occur both at the individual and contextual level, including governance policy environment that influences health services availability.^17^ Andersen argues that factors associated with the tendency to use health services (ie, predisposing factors), availability of health services (ie, enabling factors), and severity of illness (ie, need factors) work together within a broader contextual setting (influenced by cultural norms and existing policy environments) to determine health service use.^17^ Importantly also these predisposing, enabling, and need factors interact with each other at the same time, in a dynamic and complex manner, and at varying spatial and politico-social scales. As such, they operate through more of a relational perspective rather than interactions within their fixed categories. The relational perspective of place emphasizes the salience of vertical linkages based on place-based politico-social relations, resulting in constantly evolving multidimensional exchanges between people with their wider environment at varying scales, which in turn influences their health outcomes and care utilization, as shown in Figure 1.^18,19^ As such, an individual’s healthcare utilization could be influenced by their age, income level, location, distribution of facilities in their community, and personal health beliefs but also the health policies enacted at the federal and operationalized at provincial level, all at the same time. For example, T2D service utilization by a 30-year-old NPR in Ontario would have been contingent on the 3-month wait period before the COVID-19 pandemic vs. after 2020 (no wait period currently), as well as other factors such as their ethnicity, educational level and occupation, personal health beliefs, gender, and out-of-pocket costs simultaneously. Likewise, due to exclusion of universal prescription drug coverage in the CHA (federal level policy), the utilization of prescription medications for a 67-year-old resident in Ontario would differ from that of a 45-year-old resident because of varying coverage offered by the province, as well as household incomes, evaluated and personal needs, health beliefs, and other factors interacting concurrently.

This study thus contextualizes relevant components of the Andersen’s BM, and combines them with the relational perspective of place to present a new conceptual framework that shows these dynamic interactions, as visualized in Figure 1 above. This adapted framework was used in forming the interview guide, and contextualizing the findings. In particular, the study used the following individual-level predisposing factors: age, gender, education, occupation, ethnicity and family history. At the contextual level, cultural norms (particularly traditional diets and physical activity levels) were also considered. Likewise, individual-level enabling factors included annual household income, health insurance status, cost-sharing/deductibles, presence of primary physician, mode of transportation, and travel and wait times to see the doctor, while contextual-level enabling factors included Ontario’s provincial health policy and its extent of coverage (OHIP), costs of medications and services (eg, tests and exams), as well as availability and distribution of health services and community resources (eg, diabetes education programs or diabetes clinics). For need factors, only perceived need and evaluated need were considered. Finally, additional outcomes such as consumer satisfaction and quality of life were also incorporated into the interview guide. Figure 1 shows the interactions between all these selected factors which in turn influence the use of health services, health beliefs, and personal health practices.

## Methodology

This study used in-depth interviews to investigate the utilization of diabetes-related healthcare services and related experiences amongst South Asian individuals (particularly those from Pakistan, India, Bangladesh, and Sri Lanka) within the Greater Toronto Area (GTA) in Ontario, Canada. Of particular focus were the target municipalities (or census subdivisions [CSDs]) of Toronto, Mississauga, and Brampton. In 2016, these CSDs had the highest proportions of immigrant populations within Ontario and Canada, as well as the highest percentages of visible minorities.^28^ Not surprisingly, the vast majority of these visible minority populations also identified as South Asians.^28^ It is important to highlight here that while the initial aim of this study was to conduct face-to-face interviews, the overwhelming escalation of the COVID-19 pandemic and the subsequent lockdown restrictions made it practically impossible. Thus, only the first interview was carried out in-person (on March 08, 2020; Ontario declared a state of emergency and initiated lockdown on March 17, 2020.) and the rest of the interviews were conducted over the telephone between October and December 2020.

It is also worth noting that regardless of the time gap between the first and the rest of the interviews, the same version of the interview guide was used, even though the pandemic restrictions had drastically changed between early March and October of 2020. This decision was motivated by 2 factors: firstly, to maintain consistency with the initial points of investigation outlined in the study (which did not pertain to a pandemic), and second, given the ongoing peak of the pandemic during the interview period, investigating post-pandemic conditions and routines for the participants was not possible. Consequently, we opted to utilize the original pre-pandemic state interview guide, while acknowledging and documenting any changes mentioned by participants in response to the prevailing pandemic circumstances.

The questions in the interview focused on the general risk perceptions and lifestyle before diagnosis of diabetes; details about diabetes-related healthcare and treatment – in terms of access to and utilization of services, medications and treatments, lifestyle changes/habits, health insurance, and challenges; outcomes, satisfaction with access to and quality of health services, and the final set of questions comprised of demographic and closing questions. The target population for the interviews comprised of migrant South Asian individuals who were either born in a South Asian country (Pakistan, India, Bangladesh, Sri Lanka, and Nepal) or had ancestral origins to these countries (ie, second generation immigrants); were diagnosed with T2D; and above 18 years of age. The rationale behind targeting multiple South Asian sub-groups was that in research, South Asians are often times grouped under the umbrella term of “minority groups” or simply “South Asians,” with only a single sub-group being investigated in actuality, and it can potentially obscure the incredible diversity in language, belief systems, cultural practices, and health behaviors of these sub-groups.^29^ Even though we attempted to account for group diversity, it is imperative to highlight an important caveat of this study, that is, it is exploratory in nature, and while it endeavored for a wider inclusion of the healthcare utilization experiences of multiple sub-groups to represent the South Asian ethnicity (a high-risk ethnicity overall), it is by no means a detailed or comparative account of these sub-groups’ experiences, which was out of the scope of this study. Instead, it provides a starting point to studying the diverse healthcare utilization experiences of migrant South Asian groups living with T2D in Ontario, Canada.

Participants in this study were recruited through a combination of purposive and snowball sampling, mainly due to the COVID-19 lockdown measures in Ontario, which had resulted in most of the potential advertising locations (religious facilities, ethnic grocery stores, diabetic education centers, and primary care health clinics) to be either closed or operating remotely at the time of data collection. The uncertain pandemic situation and subsequent waves of rising COVID-19 cases throughout 2020 presented a challenge in obtaining a more diverse sample of participants as the original recruitment strategy had to be heavily amended to conform to changing pandemic restrictions.

More specifically, 2 ethnic grocery stores in Mississauga, 1 Indian/Punjabi and the other Pakistani, were requested to display the recruitment poster near their cash counters. Additionally, the authors utilized their personal and professional networks to disseminate the study. The recruitment poster was also published on Quercus, the online Canvas platform for University of Toronto, within relevant geography courses. Notably, 15 participants were recruited through network referrals, 3 via Quercus (students’ referrals), and 2 through subsequent referrals from participants. Regrettably, the altered strategy did not yield Nepali participants, rendering the study’s results non-applicable to this subgroup.

Participation in this research study was completely voluntary and interviewees were asked to read and sign a consent letter via email or provide oral consent before the commencement of each interview. Ethics approval for the research was obtained from the University of Toronto Research Ethics Board. Those who participated in the interviews were compensated for their time with a gift card of $40 CAD each, and it was communicated to them that they would not be required to return it in case of withdrawal from the study. The interviews took place over the telephone and were scheduled according to the availability of the participants. The interviews were audio recorded using an app called “Voice Record” on an iPad. All interviews were conducted in English, with the exception of 3 interviews, 1 of which was in Urdu (translated by 1 of the authors during transcription), and the remaining 2 were in Tamil for 2 of the Sri Lankan participants. For the latter 2 interviews, a family member of each participant kindly agreed to translate questions and responses to facilitate the interview. The final sample size (ie, 20) was based on thematic saturation, availability of respondents, and pragmatic considerations of the project (in light of the COVID-19 pandemic).

The audio recordings were then transcribed manually using MS Word and imported into NVivo 12 for thematic analysis and coding. A flexible coding technique was adapted, as outlined by Deterding and Waters.^30^ This technique allows the researchers to streamline the coding process, elaborate on concepts and connect them more meaningfully to the data rather than the time-intensive process of line-by-line coding in traditional grounded theory process.^30^

Flexible coding comprises of 3 types of codes: attributes, index codes, and analytical codes.^30^ The first step involved *indexing* or index coding, where each transcript was read and re-read carefully, and larger chunks of text were assigned into 15 smaller codes based on emergent themes and research questions covered by the interview guide. After the first round of indexing, transcripts were re-read to locate any responses that might have been naturally intertwined in the conversations rather than given as direct answers to questions.^30^ The authors also created memos during these multiple readings to develop a rough map of the “story” in the data. During the second step, index codes (and the relevant portions of transcripts within them) were analyzed and refined, along with the memos, and they were grouped into 5 analytic codes (or more accurately “nodes”), which represented the broader emergent themes in the interview data.^30^ Lastly, transcript files were also assigned “attributes” based on the demographic variables collected from the respondents (eg, age, gender, occupation, country of birth, length of time in Canada, occupation and so forth). These attributes were then utilized and incorporated in the analysis stage while comparing responses regarding different questions. For example, age was taken into account for healthcare coverage related questions and responses as the provincial health coverage policies vary between different age groups.

## Results

Thematic analysis of the interviews revealed 5 main themes (derived from the 5 analytic codes), categorized as lifestyle factors, health-related behaviors, diabetes management, provincial healthcare coverage for diabetes-related medicines and tests, and suggestions for improvement in the healthcare system. The respondents consisted of 10 males and 10 females in varying age groups, with the average age being 56 years. All participants were born outside Canada and had immigrated to the country as adults. Nearly half (9) of the participants originated from Pakistan, 5 from India, 3 from Bangladesh, and 3 from Sri Lanka. Most of the participants were located in a range of places within the GTA, for example, Mississauga, Brampton, Scarborough, Markham, and Richmond Hill. 17 of the participants were diagnosed with diabetes after moving to Canada, whereas 3 were diagnosed before moving to Canada, with the average duration since diabetes diagnosis being 10 years. Likewise, participants’ reported length of residence in Canada ranged from 1.5 years to 50 years (average being 17.5 years). Demographic characteristics of participants are presented in Table 1 below. Lastly, pseudonyms have been used to maintain the anonymity of participants.

**Table 1.** Summary of Participants’ Sociodemographic Characteristics.

Sociodemographic characteristics	Number of participants (%)
Ethnicity	
Pakistani	9 (45)
Indian	5 (25)
Bangladeshi	3 (15)
Sri Lankan	3 (15)
Gender	
Female	10 (50)
Male	10 (50)
Age (years)	
36-40	1 (5)
41-45	1 (5)
46-50	6 (30)
51-55	1 (5)
56-60	5 (25)
61-65	3 (15)
>65	3 (15)
Highest level of education achieved	
High school or below	2 (10)
Bachelors/college	12 (60)
Masters and above	6 (30)
Diagnosed before or after migration to Canada	
Before	3 (15)
After	17 (85)
Duration of time in Canada (years)	
<5	3 (15)
5-10	2 (10)
10-15	3 (15)
15-20	7 (35)
>20	5 (25)
Duration of diabetes since it was diagnosed (years)	
<3	3 (15)
3-6	2 (10)
6-9	4 (20)
9-12	3 (15)
12-15	5 (25)
15-18	1 (5)
18-21	2 (10)

### Lifestyle Factors

In terms of lifestyle factors, prior sources of stress, such as moving to Canada, starting all over, hectic/demanding jobs, and balancing work and family life affected participants’ ability to take care of their health in the time leading up to their diagnosis. For example, 1 participant commented:

“*We moved here and then the cultural shock and everything was on me. [. . .] I was also a teacher back home, but here I was zero, I could not get the job anywhere [. . .] so I took admission in the college also, so part-time studying, plus 4 children, plus financial problem and so many things. And my husband wasn’t very comfortable here, because he was an engineer back home and here he had to do security jobs*.” – *Maryam (RP4), Female*

In connection with this, participants mentioned how their meal schedules were irregular, that they were often working long hours, and generally not able to take care of their diet or do physical activity.

*“We start temporarily and we get stuck and then it was long hours. 12 hours every day or 24 hours every other day [. . .] and it’s stressful in the traffic. And with irregular eating habits, eating junk food most of the times, and lots of coffees at that time.” – Abdul (RP9), Male*

Abdul’s situation demonstrates a combination of the above-mentioned factors. He was a former engineer who was unable to enter the engineering workforce after arriving in Canada, and he got into taxi driving as a temporary job, which later became his permanent job. He explained how his lifestyle deteriorated due to this situation, and the frustration of not being able to work in his field also led him to start smoking and drinking heavily while already being a cholesterol patient.

Another important source of stress mentioned by some of the participants was having to balance work and family and not finding time to look after themselves, irrespective of having moved to Canada or not. This is illustrated in the comment below:

*“As a working woman, you do not take care of yourself because you have a husband, children, a job, a career, so you’re too busy trying to reach all these goals and satisfy everybody around you, and that brings on the stress. So, for years the stress was there but I didn’t know I was diabetic.” – Amrita (RP1), Female*

Many participants also had a family history of diabetes, though most did not take this risk into account in terms of making changes in their lifestyles, and some did not get a chance to make lifestyle changes as they were diagnosed at a much younger age compared to their parents. For instance, 1 participant said:

*“I have a family history, my dad, but he got diagnosed at the age of 68, but I was 47, 48. So I wasn’t aware that at this age I will get it. Mentally, I wasn’t prepared for that.” – Maryam (RP4), Female*

Furthermore, several other participants who were either aware of their family history, or were at the borderline stage, or a combination of both, did not take any steps to change their diet or physical activity levels. This is highlighted, for example, in the following quote:

*“2 years before the diagnosis, doctor told me that you can get diabetes because you are on borderline. And then I forgot, I didn’t care about that.” – Ayesha (RP16), Female*

With regard to diet and physical activity, a majority of participants mentioned being used to traditional diets and low levels of physical activity before their diabetes diagnosis. These included foods like wheat *roti* and *chapati*, chicken, meats, *daal*, oily foods, occasional traditional sweets, and most commonly rice—a staple food across Pakistani, Indian, Bangladeshi, and Sri Lankan households. While most people implied that their traditional diets were usually simple, a few others spoke openly about having an unhealthy diet. For example, 1 person said:

*“It was a lot of, lot of oil [. . .]. Today, I would say it was not a good diet. Overly spicy, and full of fat maybe.” – Rafeeq (RP3), Male*

Aside from cultural foods, a few participants also brought up the issue of unhealthy diets in terms of their dependence on junk foods for convenience purposes, and this is illustrated in their comments below:

*“Also the diet change, here everything is sweet, sweet, sweet. Because I didn’t have time to cook so much, so usually McDonald’s, Tim Hortons, all these junk foods. Sometimes only a bag of chips, and sometimes fries, and these type of things.” – Maryam (RP4), Female*

*“Before I was eating too much pizza, McDonald’s, Harvey’s, fries. . .too much.” – Faizan (RP11), Male*

With regard to an active lifestyle, more than half of the participants mentioned not doing regular exercise or physical activity before they got diagnosed. One main reason for this was not having enough time or energy to do much exercise after a long day at work, especially if one was standing or walking all day long, as mentioned in the comment below:

*“I am in retail, so I have to stand all the time. If I work 8 hours, almost 7 and half hours I will be standing. So I don’t have stamina to do physical exercise after that.” – Aarush (RP7), Male*

Other than this, participants either viewed exercise as unimportant, or they considered walking and day-to-day work to be sufficient physical activity. One of the participants stated:

*“Actually I’m not doing any exercise still. Just, sometimes I walk 3-4 kilometers in a day, every day. That has been my exercise all my life.” – Saad (RP12), Male*

It is interesting to note that participants reported that there were significant changes in their dietary and exercise behaviors after their arrival in Canada and before their diagnosis. Although participants admitted that they continued to consume less healthy food options from their origin countries, they also indicated that they incorporated other western diet options that are high in sugar, salt, and fat. While many indicated that opportunities for keeping active exist within their locations of residence, their busy work schedules made it difficult to exercise regularly.

Nevertheless, these aspects were consciously improved and reformed by them after diagnosis, whether that be through dietary choices, physical activity, or a combination of both. When asked whether they found it challenging to make these transitions, a majority of participants admitted that it was hard for them to adjust at the beginning, especially since they were used to specific kinds of foods all their lives and cutting them out completely was difficult. 1 participant (Amrita) explained this in her comment below:

*“You know I was 65 years old. . . I had been used to a certain way of eating certain tastes and flavors and now all of a sudden, I couldn’t have the hot rolls with a lot of butter and this was the challenge, how to replace it.” – Amrita (RP1), Female*

Even though these changes were hard at first, most people explained that they were now very careful and strict with their diet.

When participants were asked whether they had consulted a dietician after getting diagnosed, 9 participants indicated that they had. Out of these, 4 had consulted with a dietician likely only once – when they were initially diagnosed in Canada and referred by their family doctor. These singular referrals took place between 3 to 9 years ago for 2 of the participants (based on the duration since their diagnoses) and as long as 12 to 15 years ago for the other 2. On the other hand, 5 out of the 9 participants indicated being in contact with their dieticians on a regular basis, that is, every 6 months. Notably, the remaining 11 participants had not consulted a dietician at all after being diagnosed. The most common reason given by participants for this was unawareness that these resources exist and how they can be accessed. This problem was explained by one of the participants in the following comment:

*“I am not aware that whether or not we can consult a dietician, who are those dieticians, and how to consult them. And would it be free or would it cost us [. . .] so that also prevents us or discourages even to seek something like this.” – Zabir (RP5), Male*

Other reasons included perceived unimportance of dieticians, participants’ busy schedules, and lack of guidance and referrals by family doctors to such dieticians. Participants also brought up the transition to a more physically active lifestyle as an important change after their diagnosis. A majority of participants mentioned incorporating some form of exercise in their daily routine, most commonly walking. In terms of challenges in this area, 1 participant voiced their concern that it was difficult to go out and walk in the winter months. Another participant indicated the financial cost of exercising as a limiting factor, that is, that gym memberships were expensive and difficult to maintain, resulting in an inability to exercise in an indoor environment regularly. Several participants also indicated that their busy schedules and associated lack of time, and more recently, being stuck indoors due to COVID-19 restrictions were important reasons for not being able to exercise. Overall, it was apparent that all participants recognized the importance of lifestyle changes after their diagnosis, however, the extent of the adoption of these changes varied with factors such as financial barriers and busy schedules acting as impediments.

### Health-Related Behaviors

Surprisingly, a majority (ie, 11 participants) of participants did not experience any signs or symptoms of diabetes and were diagnosed through annual blood checkups or incidentally, as illustrated in the quote below:

*“I never developed any symptoms like excessive thirst or excessive hunger or extra times to go to washroom or toilet, nothing like that. I never had any feeling that I have diabetes or any problem is going on inside me.” – Hamza (RP13), Male*

For the 9 participants that did experience symptoms, there were mixed responses in terms of how quickly participants sought medical help. Some waited till their symptoms got worse, while others contacted their doctor immediately. The initial reactions of participants to their diabetes diagnosis (ie, worry, stress, anger, and indifference) also shaped how they came to terms with their condition and made changes to their lifestyle. A few participants explained that it took some time for them to come to terms with this situation, as exemplified in the quote below.

*“[. . .] I know, whoever has diabetes, has to maintain their food habit. Maintain the medication all the time. That’s why I was pretty upset. [. . .] I never want to take any medication.” – Saad (RP12), Male*

The initial perceptions of diabetes being a severe disease also prompted a few to do their own research on the condition, or sign up for short term diabetic classes as well. The following quote highlights this sentiment:

*“I started reading a little bit about it and went for classes with the diabetic centre and got quite a bit of information, that it is not the disease that is dangerous, it’s the complications.” – Amrita (RP1), Female*

Hence, there was a mix of reactions, with some participants being more optimistic than others. One of these participants mentioned that she continued to eat unhealthy diets for the first 2 years after her diagnosis, which highlights her denial of the situation. The fear of risks and complications of diabetes was acknowledged by most participants and that in turn compelled them to take greater care in their diabetes management, keep up with the related tests and exams, and generally become more attentive to their health compared to before they got diagnosed. However, it is worth noting that there were variations in how the participants themselves kept up with and utilized these tests and exams. Some participants spoke about being very thorough, that is, getting their blood work done every 3 months and seeing a few specialists once a year (such as for eye or feet examinations) as per requirement. For example:

*“I see about 4-5 doctors in a year, specialists. [. . .] I never miss my appointment and go regularly to see my eye doctor. I am very particular about it.” – Rafeeq (RP3), Male*

Others were simply maintaining regular blood and eye exams and considered them to be sufficient. Even with these variations, generally, all participants communicated their active efforts to keep up with their required tests.

### Diabetes Management

When it came to diabetes management, particularly medications and equipment, metformin was the most frequently mentioned form of medication, while insulin was used by only a few participants. When asked whether their doctor or healthcare provider had explained how to take these medications, 10 of the participants affirmed that their doctor had provided proper guidance in person. However, 6 participants also brought up the fact that their doctor only wrote a prescription, and that it was the pharmacist that explained about the medication. This is exemplified by the following quotes:

*“[. . .] doctor didn’t tell me any side effects, she just prescribed the medicine. And the pharmacist gave me all the instructions on how to take them. But also, pharmacist didn’t tell me about the risks and side effects.” – Ayesha (RP16), Female*

*“They just wrote a prescription, no clear advice, except just take it in the morning and evening.” – Zabir (RP5), Male*

When discussing equipment to self-check blood sugar, glucometer and test strips were commonly mentioned by a majority of the participants, although a newer device consisting of a sensor patch (the Freestyle Libre) was also highly praised and preferred by a few participants, as indicated in their quotes below:

*“You can see and monitor [. . .] every time after eating, or if I’m eating anything wrong. Immediately I can see. So it’s much better, and here on my phone I can see everything is recorded. So it’s very helpful.” – Bhavna (RP8), Female*

*“I prefer the reader, very easy to use, you can use it anywhere without taking blood from your finger and that helps quite a bit.” – Amrita (RP1), Female*

Even though this is a helpful device, its knowledge and uptake seemed to be quite low among the respondents, as only 3 mentioned using it. These 3 participants had varying durations since their diabetes diagnosis (ranging from 7 to 14 years) as well as varying lengths of time since arrival in Canada (ranging from ~7 to 50 years), so the knowledge of this new technology did not seem to be correlated to duration of diabetes or time spent in Canada. However, 2 of these participants also expressed concerns about the cost of the sensors. It is important to note that both participants who voiced the issue of sensors being expensive came from different brackets of annual household income (ranging from CAD $60 000-$70 000 and $90 000-$100 000), which highlights cost as a potential limiting factor across a range of income brackets, especially lower ones. Family doctors also played an important role in diabetes management, as they are the ones prescribing or adjusting medications, ordering tests, referring to specialists, and so forth. Almost all of the participants expressed their satisfaction with their family doctors, both in terms of their interactions/communication and the management of their condition. The accessibility of family doctors contributed as an important aspect of participants’ overall satisfaction. Nearly all participants resided within the same city as their family doctors, and were within easy reach, that is, a maximum of 10 to 20 min driving distance, usually even less. However, there were varying views amongst the participants on waiting times. Additionally, participants also displayed a high level of satisfaction with the kind of technical care they received, such as blood samples being drawn, how thorough eye and foot exams are, etc.

Beyond medications and tests however, participants did indicate a need for family doctors to play a more active role in informing diabetic patients about additional resources within the community (eg, diabetic centers, clinics, seminars, and community education programs). The Trillium Diabetic Centre in Mississauga is a prime example of one such facility, and only 4 participants mentioned using this facility and seeing the specialists there. All 4 of these participants were retired individuals, aged above 60 years, and had been diagnosed for over 10 years (in some cases almost 20 years). The rest of the participants had varying reasons for not using such resources, frequently citing unawareness that diabetic clinics or centers exist in their communities, as exemplified in the quote below.

*“I am not aware of that. [. . .] my GP knows very well and he must have informed in the system that I am a diabetic person. So I did not get any pamphlets or any updates [. . .] that something is going on in our community, or somewhere near, which I can go and attend or something. So how can I come to know?” – Hamza (RP13), Male*

This lack of awareness was voiced by participants regardless of how long they had been living in Canada (ie, from less than 3 years to over 20 years). It is important to note that being able to use these resources also depends largely on the family doctor, as pointed out by one of the participants. For example, a referral is required from the family doctor in order to avail services offered by the Trillium Diabetic Centre. This is highlighted in her quote below:

*“Unfortunately, this has been my gripe that it is not easy to access. It’s just by chance [. . .] that these resources came to me through my doctor who put me onto the diabetic specialist at Trillium Diabetic Centre. Otherwise, I didn’t have a clue that such facilities existed. You cannot avail these facilities unless your doctor recommends it, and this is where the system fails.” – Amrita (RP1), Female*

Aside from the issue of awareness, there were also perceptions of unimportance in relation to using these resources. For example,1 participant stated:

*“I don’t need that because I get that literature on my emails and everything [. . .] about the diet, how to live with diabetes, I regularly get these type of emails.” – Maryam (RP4), Female*

Generally, it was apparent that very few participants used resources like diabetic clinics or centers to supplement their diabetes management, whereas the majority were either unaware of these resources or unwilling to use them.

### Health Insurance Coverage

An important theme that emerged was the varying healthcare coverage available to the participants for their diabetes medicines. To recount, OHIP does not cover any prescription medicines for individuals aged over 24 and under 65 years, and they have to commonly rely on either the Trillium Drug Program or the Ontario Drug Benefit Program.^24^ In the case of the study participants, some were paying for their medicines entirely out of pocket, some were only partially covered, and a few were fully covered. Participants in the older age groups (above 65) reported better coverage (under the Ontario Drug Benefit Plan), relative to other age groups. A considerable number of participants (ie, 16) were under 65 years, so their drug coverages were fairly different. Among these participants, a few had company insurance from their jobs, some were paying partially out of pocket and were partially covered by the Trillium Drug Program, and the rest were handling the expenses entirely from their personal funds. People especially in the latter 2 situations frequently expressed their dissatisfaction with the healthcare coverage during the interviews, indicating that the price was too high for their medications and that as diabetic patients they should be getting more coverage. . These views are illustrated in the following quotes:

*“I have to pay lot of money. . .lot of money. They don’t cover everything, so I have to pay a certain amount.” – Maryam (RP4), Female*

*“I am not satisfied at all. This is too high of a price for a medication, particularly for a condition like diabetes, but obviously as a patient, I don’t have a choice, I have no choice but to pay for it.” – Zabir (RP5), Male*

Some participants also suggested that diabetic medications should either be provided for free or at a nominal charge to the patients as this is a long-term illness. One person even highlighted that it would be economically more feasible for the government to invest in providing free diabetic medications rather than dealing with the complications that arise in patients as a result of them not taking medications due to financial constraints.

*“As Canada is a welfare state, [. . .] they should take care that anyone who has been formally diagnosed, they should be given these medications, either free or at a very nominal charge, but it’s not happening. We’re paying full amount for the drug on the counter, and it really stings.” – Hamza (RP13), Male*

This discontent also seemed to come from the fact that even with the Trillium Drug Program, coverages were vastly different from person to person, and there was little to no information available to people in terms of how this program works and how their out-of-pocket costs are calculated. As a consequence, some participants were paying more than the others based on their annual income. Even with these varying coverage issues, all participants expressed that they were diligent about regularly getting their diabetes medications, as it was important for their health. This perspective is expressed in the following quote.

*“Even if it is expensive and everything, I am committed that I have to take the medication. At no point I thought that I wouldn’t buy medication.” – Abdul (RP9), Male*

Participants’ overall thoughts on the provincial health insurance and how it covers diabetic health services and tests were also mixed. While some were satisfied with the current system, others explained that it does not provide feasible coverage for everyone, especially those who cannot afford the expensive medicines, insulin, test strips, or the patches, as illustrated in the following quote:

*“For those who do not have any coverage with the company that they’re working in, and don’t have private insurance, this is a huge problem. That’s why we have so many diabetics, because they’re not taking being taken care of, they have to pay, and that is a lot of money.” – Amrita (RP1), Male*

Most importantly, there was a general lack of awareness regarding how the provincial healthcare coverage works for diabetic patients specifically, including not just the Trillium Drug Program (for medicines), but also coverage for eye exams, foot exams, glucose test strips, and so forth. Here too, participants indicated a need for family doctors to better inform them about what is covered for them as diabetic patients and what is not, as highlighted in this comment below:

*“I don’t know about provincial diabetes plan or something like that. [. . .] If you go to your family doctor, they will not mention these kinds of plans. They will tell you about bloodwork, they will give you a prescription, but they don’t mention any kind of special coverage for diabetes.” – Ridhi (RP17), Female*

Participants that alluded to this lack of knowledge included those who had been living in Canada both for a short time and long time (ranging from approximately 3 years to almost 30 years). Likewise, their diabetes duration also ranged from less than 2 years to 20 years, so this issue did not seem to be a result of inexperience in Canada or unfamiliarity with disease management alone. The lack of knowledge about provincial health coverage left several participants unsure of what services were covered for them, and how they could avail them without having to worry about the associated costs. One participant even spoke about relying on health check ups and tests in India when she travels there as it is less costly and easier for her get the specialist appointments over there than in Canada.

### Suggestions for Improvement

Participants also provided general suggestions to better address diabetes in the healthcare system, such as increasing education and awareness about diabetes for those at higher risk and younger generations:

*“If the patient is really in trouble, or facing something, and doctors know that it’s going to link with something dangerous, they should be more responsible, and they should be telling us where to go. So I think it should be more on display, [. . .] in media and even doctor’s clinics also.” – Ridhi (RP17), Female*

This particular issue was also highlighted when participants were asked whether they had received any health information about diabetes and its risks and management since coming to Canada, and many had responded that they had not. Several people said that they received it from their doctor, or they did their own research on the internet, but only after their diagnosis. Participants also mentioned that receiving information and newsletters about dietary guidelines would be helpful for many patients, especially if cultural differences in traditional diets are acknowledged and presented accordingly.

*“I think it would be nice to educate people, for sure. Maybe through some newsletters, explaining to people what to see. [. . .] Like different cultures have different types of food, maybe tell them what’s benefitting their health, how they can control.” – Ishani (RP10), Female*

The absence of knowledge regarding additional facilities and seminars available in the communities also relates to this theme. Likewise, the lack of information on how to properly avail healthcare benefits for diabetic patients was also emphasized by the participants. They explained that family doctors can play a better role in informing them on how to access health services for diabetic patients, as exemplified in the following quote:

*“When we go to our GP or doctors, and they come to know that we are diabetic, they should explain to us that these are the free things available for us. Or these are the benefits we can check for, and whether we can get those benefits or not.” – Aqsa (RP19), Female*

Finally, 1 participant also spoke about the need to keep diabetic patients up to date on the latest research being done on diabetes, insulin, new diabetic medicines, and so forth, as it gives them hope for the future.

*“I think diabetes is not a curable disease, it is lifetime. So, we need some lifetime education, as new medications come in, maybe new insulin, I think we should be informed. [. . .] It gives us hope [. . .] so we can look ahead and say ‘well, right now I’m suffering but my children may have a better time.’ So I think education, we have to have more, we don’t have enough.” – Amrita (RP1), Female*

Overall, the suggestions emphasized the importance of awareness efforts that need to be taken up in the healthcare system in order to improve the lives of existing diabetic patients and reduce future cases of this disease.

## Discussion

This study examined how South Asians living with T2D utilize diabetes-related health services within the GTA, Ontario. The findings indicate dissatisfaction among respondents regarding the current healthcare coverage and drug plans provided by Ontario for diabetic patients, especially those under the age of 65 years. This issue of financial barriers and out-of-pocket costs impeding diabetes management has been well documented in the literature.^26,31^ The current study shows that even those with partial drug coverage face financial strains owing to co-payments or deductibles, and this in turn may influence their healthcare decisions. In other words, these are enabling factors that play a significant role in influencing access to and utilization of recommended diabetes care. Simultaneously at the individual level, these can be associated with respondents’ income level, status of healthcare coverage, and out-of-pocket costs or deductibles, which in turn shape their ability to get prescription medications as well as testing supplies for example, glucose strips. Enabling and predisposing factors such as age, ethnicity, depression, and involvement with treatment decisions were also found to be associated with the predictive underuse of medicines by patients in a comparative study by Kemp et al.^32^

Additionally, even though all participants perceived diabetes-related tests and exams to be important, there was considerable variation in terms of how each participant kept up with these tests, which ultimately leads to the unequal and sometimes under-utilization of necessary services. This could be associated with different levels of perceived need among participants, different locations of care (enabling factor), unequal awareness and knowledge levels among participants (predisposing factor), as well as varying monitoring and advice practices among family physicians (evaluated need factor). Even so, the difficulties with navigating a complex healthcare system, especially as an immigrant, are consistent with previous literature.^21,22,26^

Relatedly, there was also variation in knowledge and uptake of diabetes-related technology such as glucometers and test strips for blood sugar monitoring, as well as newer forms of monitoring equipment such as the Freestyle Libre sensor patch and reader. This further points to differences in knowledge and uptake of such devices, and could be related to financial barriers, as the sensor patches were considered expensive even by the few participants who used them. Financial burdens have been frequently highlighted within previous studies in terms of preventing diabetic patients from obtaining necessary medical supplies and devices.^26,32^

With regard to diabetes treatment and management, one of the main findings was that there was a high level of satisfaction among study participants with the diabetes services they used (eg, blood tests, eye exams, and foot exams), as well as with their primary care or family physician within the GTA. We also did not find any allusion to communication difficulties in the overall interactions between the with healthcare providers and participants, contrary to existing literature in this regard for South Asian patients.^20-27^ This could be attributed to several enabling and predisposing factors, such as close geographical proximity to their physicians, ability to receive appointments easily, some primary physicians being either bilingual or of similar ethnic background (as alluded to in participants’ responses), and most of the respondents being fluent in English and well-educated (with the exception of 2 Sri Lankan participants). However, the small sample size suggests it would be unwise to generalize this level of satisfaction to all diabetic South Asians within the GTA.

Our study also highlights that while family doctors are playing a reactive role in managing diabetes, they fall short in terms of providing proactive advice to South Asian patients, that is, in terms screening for early signs of diabetes and increasing health literacy about this condition, as reflected by the fact that many respondents received their diabetes diagnoses incidentally and unexpectedly. This could partially be attributed to absence of signs and symptoms among many of the participants, but also a lack of rigorous screening measures by family physicians, especially for high-risk South Asian patients. This points to a need for health professionals to educate not just South Asian patients, but other high-risk patients as well, and to develop culture-specific collaborative approaches for diabetes management, which has been actively emphasized in previous research.^3,33^ Doing so could also potentially improve patients’ perceived health needs so that they may be more attentive to their health and seek care immediately upon noticing any signs or symptoms of diabetes.

In connection with utilization of additional diabetic resources (such as diabetic centers, diabetic clinics, or diabetic education programs) there was considerably low uptake among the participants. When considered from a relational perspective, one would expect this issue to be a result of various intertwining predisposing and enabling factors, such as conflict between work schedules and centers’ hours of operation, patients’ self-efficacy in managing their diabetes, lack of interest, forgetfulness, long distance to a center, or a general unfamiliarity with such resources, as was the case in the findings of Gucciardi et al^34^ in a Toronto-based study. However, locational distance or work-schedule conflicts were not among the factors cited by participants in the current study for their under-utilization of additional diabetic resources. Rather, it was largely due to unfamiliarity with such resources in their communities (ie, a predisposing factor), which in turn could be stemming from several predisposing factors, for example, difficulties with navigating the healthcare system, personal health beliefs, education, occupation, levels of disease awareness, and so forth. This finding correlates with a recent UK-based study, where South Asian participants indicated lack of awareness as the main reason for not attending any diabetes programs in their community, despite availability of such programs.^3^ This finding also underlines organizational and provider-based barriers (contextual enabling factors), in terms of lack of adequate information provided by physicians to patients about such resources and encouraging them to utilize them.^3,22^ Put simply, optimal use of health services is a reciprocal process, necessitating both patient knowledge and skills, but also willingness from physicians to support the effective use of community resources.

### Limitations

As is the case with all research, there were limitations to this study as well. Firstly, due to the small sample size, the findings may not be generalizable to all South Asians (or even the 4 sub-groups investigated in this study) within the GTA, as it is comprised of many diverse municipalities (including urban, suburban, and rural areas) and different distributions of health facilities, primary physicians, and South Asian sub-groups, thus leading to varied experiences based on a number of these factors. The intention of this study was to provide a starting point to explore the healthcare utilization experiences of diabetic South Asians located within the GTA. The findings identified above can be verified in future studies focusing on specific South Asian sub-groups with larger sample sizes.

Second, participants were primarily recruited through purposive and snowball sampling, which may have introduced a sampling bias in the study. However, the study also highlights the efficacy of snowball and purposive sampling in recruiting respondents from hard-to-reach populations, as posited in previous literature. An additional limitation was that interviews were conducted in English only, therefore other potential respondents may have been overlooked on the basis of their limited English-speaking abilities. These respondents could have provided a wider range of experiences, including insights on language barriers and communication difficulties when accessing healthcare services. However, due to the small scope of this study, diverse language-range within South Asian groups, and the restrictions posed by the COVID-19 pandemic, it was unfeasible to hire translators to accommodate such potential participants.

Despite these limitations, this study is valuable in providing an exploratory account of South Asian patients’ experiences of utilizing T2D health services and resources in the GTA, Ontario. It presents perspectives from participants with diverse backgrounds in terms of education, occupation, age (ie, above vs. below 65 years), duration since diabetes diagnosis, and duration of residence in Canada. It is interesting to note that views regarding dissatisfaction with the healthcare coverage or unawareness of diabetes resources for example, were similar across all these diverse participants, indicating that these problems are not just unique to recent or less educated immigrants but in fact a reflection of the deep-set systematic barriers present within the healthcare system and related organizations that prevent diabetic South Asian individuals from making full use of the services available to them.

### Policy Implications and Recommendations

The findings of this study indicate a scope for improvement at both the provider and organizational level. First, physicians and pharmacists need to increase their knowledge of the extent of provincial coverage available to diabetic patients, drug plans, and other available resources for patients who face drug coverage issues (eg, referrals to social workers or diabetes educators).^32^ Second, physicians need to move away from practice culture in their decision-making and be cognizant of financial barriers when prescribing medications in order to reduce out-of-pocket costs, for example, less expensive generic medications, and connecting patients to other resources such as social workers or patient navigators.^31,32,35^

Policy makers and politicians also need to seriously address the glaring weakness of our healthcare system that is the absence of a universal pharmacare plan in Canada. A lot has changed since the *Canada Health Act* was first introduced in 1984, and prescription drugs play a much more critical role in improving health today than they did back then.^36^ The costs associated with these drugs have also skyrocketed over the years, putting the whole system at risk of becoming unaffordable, a situation that is now a reality for many subsets of the population.^36^ Canadians have to pay for prescription drugs “through their taxes, through their premiums, through their wages, and then they pay some more when they reach into their pockets to cover their copayments or deductibles.”^36^(p. 55) This issue has been overlooked for the past 60 years by Canadian policymakers, despite numerous recommendations from national commissions and government reports, as well as robust cost proposals.^23,37,38^ It is only recently that pharmacare has gained attention within the public and political discourse, as demonstrated by the establishment of the Advisory Council on the Implementation of National Pharmacare by the federal government.^38^ The federal government needs to restructure and replace the haphazard patchwork of public and private drug plans in its provinces with a streamlined pharmacare plan as a priority, because we cannot claim to have a universal public healthcare system if it excludes the very medications necessary for preventing and treating illnesses. It is high time that drug coverage is provided for all individuals, regardless of their age, income, ethnicity, or social status. This is crucial to ensure a more equitable healthcare system in Canada.

In terms of utilization of health services, primary care physicians need to take more initiative in enhancing equitable access to additional community resources (eg, diabetic clinics and management programs) and facilitating awareness and utilization of these resources among ethnic minority patients. Likewise, community diabetic programs and organizations need to improve their outreach efforts, especially for minority groups who usually experience additional language and literacy barriers and are unable to make full use of the services available to them. These recommendations are also transferable to other high-risk groups and ethnic minorities because barriers to access and utilization of health services are often shared across minorities, and these recommended changes could bring about a positive change for all diabetic patients and those with other chronic conditions.

## Conclusion

The findings of the study reflect differences in utilization and uptake of diabetes related health services among migrant South Asian diabetic patients residing within GTA, Ontario. . While some predisposing and enabling factors may play a role in impeding equitable access for this particular group, it is also clear that some broader enabling factors (eg, organizational and provider-based barriers, provincial health policy) at varying government levels ultimately contribute more toward disparities in access and unequal utilization of health services (eg, varying screening and monitoring practices, inadequate information provided to the patients, lack of prescription drug coverage, and lack of referrals to additional community resources).

Canada is home to numerous cultures and ethnicities, and its universal healthcare system is one of its strong pull factors in attracting immigrants from all over the world, however, it is also the only developed country with a universal healthcare system that does not cover prescription drugs. This study has demonstrated that even in a healthcare system with universal coverage, disparities exist, particularly for ethnic minorities, and their utilization of healthcare may be constrained due to organizational and financial barriers. Diabetes is a life-long chronic illness, and as such the support for this condition through the healthcare system should also be continuous and long-term, unbarred by age or income restrictions. This is crucial to achieve equitable access for all, and to ensure that immigrants who come to Canada in good health remain that way, or at the very least, do not go through health-related deterioration over time.

## Supplemental Material

sj-docx-1-jpc-10.1177_21501319241240635 – Supplemental material for “The Critical Services Are Out of Reach”: Diabetes Management and the Experiences of South Asian Immigrants in Ontario Supplemental material, sj-docx-1-jpc-10.1177_21501319241240635 for “The Critical Services Are Out of Reach”: Diabetes Management and the Experiences of South Asian Immigrants in Ontario by Minal Waqar and Vincent Z. Kuuire in Journal of Primary Care & Community Health
