# Supplementary material for: “The Critical Services Are Out of Reach”: Diabetes Management and the Experiences of South Asian Immigrants in Ontario
Source: J Prim Care Community Health. 2024 Mar 24;15:21501319241240635. doi: 10.1177/21501319241240635 (PMC10962024; doi:10.1177/21501319241240635)
Supplement: sj-docx-1-jpc-10.1177_21501319241240635 – Supplemental material for “The Critical Services Are Out of Reach”: Diabetes Management and the Experiences of South Asian Immigrants in Ontario [file sj-docx-1-jpc-10.1177_21501319241240635.docx]

**In-depth Interview Guide [FOR REVIEW PURPOSES ONLY]**

**Name/ Pseudonym: ___________________ Date: ________________ Type: ____________________**

**Perceptions and diagnosis of diabetes**

1. Tell me about the period just before you got diagnosed with diabetes. How did you feel?

- **Clarification (if required)**: were there any specific stressors in your life at that time? E.g. a particularly difficult phase in job/family/relationships/studies etc. that prevented you from taking care of yourself?
- **Follow up**: Did you get diagnosed after moving to Canada or before?

--------------------------------------------------------------------------------------------------------------------------------------------------------------------------------------------------------------------------------------------------------------------------------------------------------------------------------------------------------------------

1. Were you aware that you had a chance of developing type 2 diabetes (e.g. through family history, physician check-ups, personal diet and lifestyle?).
2. If so, did you take any steps to reduce your risk (dietary changes, physical activity etc.)?

- **Follow up:** What did you usually eat before your diagnosis?
- **Follow up**: Did you have an active lifestyle? i.e. did you exercise regularly?

------------------------------------------------------------------------------------------------------------------------------------------------------------------------------------------------------------------------------------------------------------------------------------------------------------------------------------------------------------------------------------------------------------------------------------------------------------------------------------------------------------------------------------------------------

1. What were the signs and symptoms that led to your diagnosis? How severe were they before you sought help?

- **Clarification (if required)/ alternate form of question**: did you notice the signs and symptoms (frequent urination, increased thirst, fatigue, slow healing of wounds, tingling or numbness in hands and feet, weight change etc.) and seek help immediately or did you wait until they got worse?

--------------------------------------------------------------------------------------------------------------------------------------------------------------------------------------------------------------------------------------------------------------------------------------------------------------------------------------------------------------------

1. How did you react after you had been diagnosed? What were your thoughts?

- **Clarification**: What was your reaction? Did you feel worried, sad, angry, neutral etc.?

------------------------------------------------------------------------------------------------------------------------------------------------------------------------------------------------------------------------------------------------------------------------------------------------------------------------------------------------------------------------------------------------------------------------------------------------------------------------------------------------------------------------------------------------------

**Diabetes care and treatment**

1. Have you received any education/health information about diabetes, its risks and management since you came to Canada?

- **Clarification**: From your healthcare provider or elsewhere?

--------------------------------------------------------------------------------------------------------------------------------------------------------------------------------------------------------------------------------------------------------------------------------------------------------------------------------------------------------

1. Are you on medication? If so, what type of medication do you take (e.g. pills, insulin, etc.)?

- **Follow up**: How do you pay for this (personal funds; private; or company insurance benefits; or OHIP)?
- **Follow up**: Are you satisfied with the cost of these medications? If not, does it deter/discourage you from taking your medications?

**---------------------------------------------------------------------------------------------------------------------------------------------------------------------------------------------------------------------------------------------------------------------------------------------------------------------------------------------------------------------------------------------------------------------------------------------------------------------------------------------------------------------------------------**

1. Has your doctor (or healthcare provider) explained how to take your medications? How did they go about this process?

----------------------------------------------------------------------------------------------------------------------------------------------------------------------------------

1. Do you use any additional equipment and supplies to self-check your blood sugar?

----------------------------------------------------------------------------------------------------------------------------------------------------------------------------------

1. Do you have any difficulties with taking or getting your medication (and additional equipment)? If so, what are they?

--------------------------------------------------------------------------------------------------------------------------------------------------------------------------------------------------------------------------------------------------------------------------------------------------------------------------------------------------------------------

1. How has your diet changed after your diagnosis? Did you face any challenges in making these changes?

- **Clarification**: what do you eat generally throughout the day? Is your diet based on ethnic/ cultural/ religious beliefs?
- **Follow up**: Have you ever consulted a dietician in Canada regarding after your diagnosis? If not, why is that?

------------------------------------------------------------------------------------------------------------------------------------------------------------------------------------------------------------------------------------------------------------------------------------------------------------------------------------------------------------------------------------------------------------------------------------------------------------------------------------------------------------------------------------------------------

1. Do you exercise regularly and do you find time to exercise regularly? Are there any challenges you face in this area?

- **Follow up**: How do you usually travel? i.e. car, public transit, walking, bicycle etc.

--------------------------------------------------------------------------------------------------------------------------------------------------------------------------------------------------------------------------------------------------------------------------------------------------------------------------------------------------------------------

1. What are your thoughts on the risks and complications of diabetes? Are you afraid of them?

- **Follow-up**: Do they compel you to take greater care in your diabetes management?

--------------------------------------------------------------------------------------------------------------------------------------------------------------------------------------------------------------------------------------------------------------------------------------------------------------------------------------------------------------------

1. How has your overall perception/attitude changed towards seeking treatment after your diagnosis compared to before you got diagnosed?

- **Clarification**: would you say that you’ve become more attentive to your health and now seek medical care more quickly if you experience any signs of complications? Or is it the same as before you got diagnosed with diabetes?

-------------------------------------------------------------------------------------------------------------------------------------------------------------------------------------------------------------------------------------------------------------------------------------------------------------------------------------------------------

1. How important do you think it is to keep track of your next exam/test (i.e. HbA1C, lipid screening, foot exam, dental exam, etc.)?

- **Follow-up**: How regularly do YOU keep up with these tests? If you don’t, why is that? Are there any challenges you face?

--------------------------------------------------------------------------------------------------------------------------------------------------------------------------------------------------------------------------------------------------------------------------------------------------------------------------------------------------------------------

1. What are your thoughts on the provincial health insurance and how it covers diabetic health services/tests?

- **Follow-up**: how much do you think that impacts or helps your diabetes management?

--------------------------------------------------------------------------------------------------------------------------------------------------------------------------------------------------------------------------------------------------------------------------------------------------------------------------------------------------------------------

1. Do you use or are you aware of any additional resources within your community (e.g. diabetic seminars, information resources, or other medical facilities)?

--------------------------------------------------------------------------------------------------------------------------------------------------------------------------------------------------------------------------------------------------------------------------------------------------------------------------------------------------------------------

1. How accessible do you think are the health facilities and these additional resources around you? Are they easily available? Would you like to change anything about them?

------------------------------------------------------------------------------------------------------------------------------------------------------------------------------------------------------------------------------------------------------------------------------------------------------------------------------------------------------------------------------------------------------------------------------------------------------------------------------------------------------------------------------------------------------

1. Have you experienced any problems/challenges with any aspect of your diabetes care (in terms of seeking treatment and managing your condition)? If so, what have you experienced? What is working well and what is not?

- **Follow-up**: what changes do you think would help with these challenges?

--------------------------------------------------------------------------------------------------------------------------------------------------------------------------------------------------------------------------------------------------------------------------------------------------------------------------------------------------------------------

1. Do you have any suggestions/thoughts you would like to add as to how diabetes can be better addressed by our healthcare system?

--------------------------------------------------------------------------------------------------------------------------------------------------------------------------------------------------------------------------------------------------------------------------------------------------------------------------------------------------------------------

**Health outcomes**

1. Tell me about where you where you seek services for the management of your diabetes (e.g. location of primary caregiver, general location of diabetes care services vis a vis current residence)
   - **Clarification**: Do you get your diabetes treatment in the same city that you live in? If it is in a different city, why is that so?

------------------------------------------------------------------------------------------------------------------------------------------------------------------------------------------------------------------------------------------------------------------------------------------------------------------------------------------------------------------------------------------------------------------------------------------------------------------------------------------------------------------------------------------------------

1. How will you describe your relationship with your healthcare provider or doctor in terms of the management of your diabetes?
   - **Clarification**: Do you have confidence in your doctor/healthcare provider in managing your condition well? Why or why not?

--------------------------------------------------------------------------------------------------------------------------------------------------------------------------------------------------------------------------------------------------------------------------------------------------------------------------------------------------------------------

1. How satisfied do you feel after your interactions/communication with your doctor and/or healthcare provider? Do you experience any difficulties? What do you like and what would you want to change?

------------------------------------------------------------------------------------------------------------------------------------------------------------------------------------------------------------------------------------------------------------------------------------------------------------------------------------------------------------------------------------------------------------------------------------------------------------------------------------------------------------------------------------------------------

1. What are your thoughts on the travel times and waiting times to see your doctor/healthcare provider?

- **Clarification**: are you satisfied with them? Or would you like to change anything?

--------------------------------------------------------------------------------------------------------------------------------------------------------------------------------------------------------------------------------------------------------------------------------------------------------------------------------------------------------------------

1. Do you feel satisfied with the kind of technical care you receive at health facilities? E.g. blood tests, eye exams, foot exams etc.? If not, why?

- **Clarification**: In terms of how the tests are done, people who take the samples/blood, etc. etc.

--------------------------------------------------------------------------------------------------------------------------------------------------------------------------------------------------------------------------------------------------------------------------------------------------------------------------------------------------------------------

1. How has your overall quality of life changed since your diagnosis

- **Clarification**: in terms of physical, and mental health, and social relationships?
- **Follow-up**: Do you think that the type of care/services you receive helps with any of these changes?

------------------------------------------------------------------------------------------------------------------------------------------------------------------------------------------------------------------------------------------------------------------------------------------------------------------------------------------------------------------------------------------------------------------------------------------------------------------------------------------------------------------------------------------------------

1. Is there anything else you would like to add or to conclude before we move on to some final general socio-demographic questions?

--------------------------------------------------------------------------------------------------------------------------------------------------------------------------------------------------------------------------------------------------------------------------------------------------------------------------------------------------------------------

**Socio-demographics**

1. Age range: ______________________
2. Gender: _____________________
3. Highest level of education: _____________________________
4. Occupation: ___________________________
5. First generation or second-generation (i.e. born outside of Canada? If so, where?): _________________
6. Length of time in Canada: _________________________
7. Household income range/bracket: ­­­­­_________________________
8. Length of time since diabetes diagnosis: _____________________

**Finish. Thank the participant.**

**Gift card preference: _____________________________.**
